# Maternal use of selective serotonin reuptake inhibitors during pregnancy is associated with Hirschsprung’s disease in newborns – a nationwide cohort study

**DOI:** 10.1186/s13023-017-0667-4

**Published:** 2017-06-20

**Authors:** Sebastian Werngreen Nielsen, Perniller Møller Ljungdalh, Jan Nielsen, Bente Mertz Nørgård, Niels Qvist

**Affiliations:** 1 Department of Surgical Gastroenterology A, Odense University Hospital, and Research Unit of Surgery, Institute of Clinical Research, University of Southern Denmark, 5000 Odense C, DK Denmark; 2 Center for Clinical Epidemiology, Odense University Hospital, and Research Unit of Clinical Epidemiology, University of Southern Denmark, 5000 Odense C, DK Denmark; 3 Engelshøjgade 26 1TH, 6400 Sønderborg, Denmark

**Keywords:** SSRI, Congenital malformation, Hirschsprung’s disease, Developmental biology, Enteric nervous system, Paediatric gastroenterology

## Abstract

**Background:**

Hirschsprung’s disease is a rare condition caused by congenital malformation of the gastrointestinal tract affecting 1:5000 children. Not much is known about risk factors for development of Hirschsprung’s disease. Two clinical cases of hirschsprung’s disease led to an investigation of the association between maternal use of selective serotonin reuptake inhibitors (SSRIs) during pregnancy and development of Hirschsprung’s Disease in the newborn child. The study examined a nationwide, unselected cohort of children born in Denmark from 1 January 1996 until 12 March 2016 (*n* = 1,256,317). We applied multivariate models to register-based data to estimate the odds ratio of Hirschsprung’s disease, adjusting for possible confounders. The studied exposure period for SSRIs were 30 days prior to conception to the end of the first trimester.

**Results:**

In the main exposed cohort the prevalence of Hirschsprung’s disease was 16/19.807 (0.08%) compared to 584/1.236.510 (0.05%) in the unexposed cohort. In women who redeemed a minimum of one prescription of selective serotonin reuptake inhibitors, the adjusted odds ratio for development of Hirschsprung’s disease was 1.76 (95%CI: 1.07–2.92). In women who redeemed a minimum of two prescriptions, the adjusted odds ratio for Hirschsprung’s disease was 2.34 (95% CI: 1.21–4.55).

**Conclusions:**

Our data suggest that early maternal use of selective serotonin reuptake inhibitors is significantly associated with the development of Hirschsprung’s disease in the newborn child. Treatment of depression during pregnancy always has to be weighed against the risks posed by untreated maternal depression. Our results have to be confirmed in other studies.

**Electronic supplementary material:**

The online version of this article (doi:10.1186/s13023-017-0667-4) contains supplementary material, which is available to authorized users.

## Background

The use of selective serotonin inhibitors (SSRIs) during pregnancy has increased significantly during the last two decades [1–3] and an increased risk of adverse birth outcomes has been reported, [4, 5] including neonatal complications [5], and groups of congenital heart and gastrointestinal malformations [4, 6–12]. Other studies have not found an increased risk of malformations [13–15].

Serotonin or 5-hydroxytryptamine (5-HT) is a monoamine neurotransmitter, which is important for the development of the enteric nervous system, and the migration of neural crest cells to the bowel wall during the first trimester [16–18]. SSRI intake during the first trimester might inhibit this process resulting in Hirschsprung’s disease (HD). HD is a congenital malformation with aganglionosis of the enteric plexuses of the bowel wall that extends from the anal canal and proximally affecting a variable length of the colon. The clinical picture is ileus with megacolon or intractable constipation, which necessitates surgery in infancy or childhood [19] resulting in severe consequences of impaired bowel function with a mixture of constipation and in faecal incontinence in early life and later [20].

During the last decade the incidence of HD has doubled [21, 22] and since only a minority of HD cases have a known genetic aetiology, further investigation into non-genetic risk factors of HD is warranted [23].

The aim of the present study was to investigate the association between maternal prescription of SSRI in the period of 1 month prior to conception to the end of first trimester of pregnancy and having a child with the diagnosis of HD. To examine this association we used nationwide Danish register data.

## Methods

### Setting

A population-based nationwide cohort study was conducted using data from Danish health registries. The uniform organization and unrestricted access to a tax-funded universal healthcare system for all Danish residents, allowed us to use a population-based study design. We used the Danish National Patient Registry (NPR) [24], the Danish Medical Birth Registry (MBR) [25], the Nationwide Prescription Database [26], and the Central Personal Registration system [27]. All registries were linked using the civil registration number. All Danish residents are assigned a civil registration number at birth or when taking residence in Denmark, and this unique identifier was unambiguously used to link data on an individual level.

### Study population

The study population included all live born children identified by the Medical Birth Registry [25] with a valid civil registration in Denmark from 1 January 1996 until 12 March 2016. By using information on the gestational age and delivery date, the date of conception was assessed for all children in the study population.

### Data sources

Established in 1977, the NPR includes records of all discharges from Danish hospitals and since 1994 all outpatient visits. Information in the NPR includes patients’ civil registration numbers and up to 20 discharge diagnoses based on the International Classification of Diseases (ICD-10 from 1994 onward) [24, 28]. The MBR consists of information on all births in Denmark since 1973 including pregnancy-related information i.e. gestational age, birth outcomes, and information on the mother, maternal civil registration number, maternal age, smoking status and parity. In 1997 the coding of maternal smoking was expanded to include possible registration of maternal smoking cessation during pregnancy. As this change was done during the study period, we reduced the complexity of the variable maternal smoking to yes (including any smoking and smoking cessation during pregnancy), no (no smoking whatsoever) [25].

The Danish Medicine Agency maintains a nationwide prescription database for all reimbursed drug prescriptions issued from Danish pharmacies. The database includes for each prescription, patients’ civil registration number, medication dosage, package size, date of reimbursement and drug classification code, according to the anatomical therapeutic chemical (ATC) classification system [29]. As only prescription drugs are registered in the Nationwide Prescription Database, over-the-counter-sale is not included. However, SSRI are only available by prescription in Denmark. The Central Personal Registration system consists of information on civil registration number, death and immigration.

### Exposed cohort, children born of women exposed to SSRI

#### Main cohort

For the children in the study population, information was collected on maternal prescriptions of SSRI (ATC: N06AB03, N06AB04, N06AB05, N06AB06, N06AB08, N06AB10) from 30 days before conception to end of first trimester (end of 12th week of pregnancy). The main exposed cohort, thus, included children of women who had filled one or more prescriptions from 30 days before conception to end of first trimester.

### Sub-cohort I

Sub-cohort I was defined as all children in the main cohort where the mothers filled one single SSRI prescription from 30 days before conception to end of first trimester.

### Sub-cohort II

Sub-cohort II was defined as all children in the main cohort where the mothers filled two or more SSRI prescriptions from 30 days before conception to end of first trimester.

### Unexposed cohort, children born of women not exposed to SSRI

The unexposed cohort constituted all children included in the study population where mothers did not fill prescriptions of SSRI from 30 days before conception to end of first trimester.

### Outcome

Outcome information was obtained from the NPR and defined as any child with a diagnosis of HD (ICD-10: DQ431) from 1 January 1996 until 12 March 2016.

### Information on possible confounders

From the Maternal Birth Registry (MBR) [25], we obtained information on the age of the mother (≤19, 20–24, 25–29, 30–34 ≥ 35 years) at the time of delivery, parity (one or more than one pregnancy), sex of the child, maternal smoking during pregnancy (yes/no), and calendar year of birth (1996–2001, 2002–2007, 2008–2011, 2012–2016).

### Statistical analysis

We constructed contingency tables for the main study variables according to the exposed and unexposed cohorts. We used logistic regression analyses to compute crude and adjusted odds ratio estimates (prevalence odds ratio with 95% confidence intervals [95% CI]) for HD following maternal use of SSRI relative to no maternal use of SSRI. These analyses were performed for the main cohort, sub-cohort I, and sub-cohort II, all relative to the unexposed cohort.

Adjustment was made for maternal age (≤19, 20–24, 25–29, 30–34 ≥ 35 years), maternal smoking status (yes/no), parity (one or more than one pregnancy), sex of the child, and calendar year of birth (1996–2001, 2002–2007, 2008–2011, 2012–2016).

### Sub-analysis

To exclude an underlying effect of other maternal disease that might be associated with malformations and use of SSRI we performed a sub-analysis. We excluded children from the exposed and unexposed cohort where the mothers had reimbursed prescriptions for any of the following medications: antiepileptic drugs (ATC: N03A), antidiabetic drugs (ATC: A10), antipsychotic drugs (ATC: N05A), anxiolytics (ATC: N05B), and tricyclic antidepressants (ATC:N06AA) 3 months prior to conception and until the end of 12th week of pregnancy. Again we used logistic regression analyses to compute crude and adjusted odds ratio estimates for HD following maternal use of SSRI, and included in the model were the same covariates as in the main analyses.

All calculations were performed using STATA Release 14.0 (StataCorp, College Station, TX, USA).

## Results

A total of 1.256.317 children were included, and of these, 19.807 children were born by women who had filled one or more prescriptions of SSRIs 30 days before conception to the end of the first trimester. Sub-cohort I included 11.351 children born by women who had filled only one SSRI prescription within30 days before conception to the end of the first trimester, and sub-cohort II included 8.456 children born by women who had filled two or more SSRI prescriptions within 30 days before conception to the end of the first trimester. A total of 1.236.510 children were born by mothers who were not exposed to SSRI 30 days before conception to the end of the first trimester. The characteristics of the exposed and unexposed cohorts are shown in Table 1, and the basic characteristics were similar according to mother’s age at delivery, sex of child and parity. Maternal smoking was more frequent in the main exposed cohort (28.6%) compared to the unexposed cohort (15.1%).

**Table 1:** Characteristics of the exposed and unexposed cohorts. The exposed cohort was divided according to number of redeemed prescriptions of selective serotonin-reuptake inhibitors (SSRI’s): minimum of one prescription, limited to one prescription, and minimum two prescriptions. Total number of children, *n* = 1.256.317

	SSRI prescriptions redeemed a minimum of one time (*n* = 19,807)	SSRI prescriptions redeemed one time only (*n* = 11.351)	SSRI prescriptions redeemed minimum two times (*n* = 8.456)	No prescriptions of SSRIs (*n* = 1.236.510)
Maternal age				
≤19	308 (1.6%)	184 (1.6%)	124 (1.5%)	18.039 (1.5%)
20–24	2.645 (13.4%)	1.530 (13.5%)	1.115 (13.2%)	145.031 (11.7%)
25–29	5.764 (29.1%)	3.234 (28.5%)	2.530 (29.9%)	415.536 (33.6%)
30–34	6.678 (33.7%)	3.779 (33.3%)	2.899 (34.3%)	437.740 (35.4%)
≥35	4.412 (22.3%)	2.624 (23.1%)	1.788 (21.1%)	220.164 (17.8%)
Maternal smoking status				
No	13.384 (67.6%)	7.838 (69.1%)	5.546 (65.6%)	946.853 (76.6%)
Yes	5.656 (28.6%)	3.095 (27.3%)	2.561 (30.3%)	186.807 (15.1%)
Missing	767 (3.9%)	418 (3.7%)	349 (4.1%)	102.850 (8.3%)
Sex of child				
Male	10.249 (51.7%)	5.862 (51.6%)	4.387 (51.9%)	601.949 (48.7%)
Female	9.558 (48.3%)	5.489 (48.4%)	4.069 (48.1%)	634.561 (51.3%)
Parity				
1	8.792 (44.4%)	4.912 (43.3%)	3.880 (45.9%)	545.646 (44.1%)
>1	11.015 (55.6%)	6.439 (56.7%)	4.576 (54.1%)	690.864 (55.9%)
Birth year				
1996–2001	1.972 (10.0%)	1.033 (9.1%)	939 (11.1%)	387.716 (31.4%)
2002–2007	6.077 (30.7%)	2.976 (26.2%)	3.101 (36.7%)	376.939 (30.5%)
2008–2011	6.787 (34.3%)	3.952 (34.8%)	2.835(33.5%)	240.952 (19.5%)
2012–2016	4.971 (25.1%)	3.390 (29.9%)	1.581 (18.7%)	230.903 (18.7%)

In the main exposed cohort we found that 16 out of 19.807 (0.08%) of the children had a diagnosis of HD, and among the unexposed cohort 584 out of 1.236.510 (0.05%) had HD, corresponding to an adjusted OR for HD of 1.76 (95% CI 1.07–2.92) (Table 2). In sub-cohort I (only one redeemed prescription), 7 out of 11.351 (0.06%) had a diagnosis of HD (corresponding to an adjusted OR for HD of 1.33 (95% CI 0.63–2.83) (Table 2). In children in sub-cohort II (a minimum of two redeemed prescriptions), 9 out of 8.456 (0.11%) had a diagnosis of HD, and the adjusted OR for HD was 2.34 (95% CI 1.21–4.55) (Table 2).

**Table 2:** Odds ratio (OR) estimates for Hirschsprung’s Disease from logistic regression models, crude and adjusted OR with 95% confidence interval (CI) for occurrence of Hirschsprung’s Disease based on all live births in the period of 1 january 1996 until 12 March 2016

	SSRI prescriptions redeemed a minimum of one time	No prescriptions for SSRIs	Crude OR (95%CI)	Adjusted OR^a^ (95%CI)
Child born with Hirschsprung’s Disease	16/19.807 (0.08%)	584/1.236.510 (0.05%)	1.71 (1.04–2.81)	1,76 (1.07–2.92)
	SSRI prescriptions redeemed one time only	No prescriptions for SSRIs	Crude OR (95%CI)	Adjusted OR^a^ (95%CI)
Child born with Hirschsprung’s Disease	7/11.351 (0.06%)	584/1.236.510 (0.05%)	1.31 (0.62–2.75)	1.33 (0.63–2.83)
	SSRI prescriptions redeemed a minimum of two times	No prescriptions for SSRIs	Crude OR (95%CI)	Adjusted OR^a^ (95%CI)
Child born with Hirschsprung’s Disease	9/8.456 (0.11%)	584/1.236.510 (0.05%)	2.25 (1,17–4.36)	2.34 (1.21–4.55)

^a^Adjusted for maternal age (≤19, 20–24, 25–29, 30–34, ≥35), maternal smoking (yes/no), sex, of child (male/female), parity (1/<1), calendar year of birth (1996–2001, 2002–2007, 2008–2011, 2012–2016)

In our sub-analysis we excluded women with prescriptions of antiepileptic, antidiabetic, antipsychotics, anxiolytic and tricyclic antidepressants, and a total of 16.395 children were born by women who had filled one or more prescription of SSRI 30 days before conception to the end of the first trimester. A total of 1.207.989 children were born to women not exposed to SSRI. The basic characteristics were similar between the exposed and unexposed (Additional file 1). A total of 13 children were born with HD out of 16.395 (0.08%), corresponding to an adjusted OR for HD of 1.76 (95% CI 1.01–3.07) (Table 3). In sub-cohort I, a total of 6 children had HD out of 9.663 (0.06%) corresponding to an adjusted OR for HD of 1.38 (95% CI 0.61–3.09). In sub-cohort II, a total of 7 children had HD out of 6.732 (0.10%), corresponding to an OR for HD of 2.32 (95% CI 1.10–4.91) (Table 3).

**Table 3:** Odds ratio (OR) estimates for Hirschsprung’s Disease from logistic regression models, crude and adjusted OR with 95% confidence interval (CI) for occurrence of Hirschsprung’s Disease based on all live births in the period of 1 january 1996 until 12 March 2016. All women with prescriptions 3 months prior to time of conception of antiepileptic, antidiabetic, antipsychotics, anxiolytic and tricyclic antidepressants are excluded from the population

	SSRI prescriptions redeemed a minimum of one time	No prescriptions for SSRIs	Crude OR (95%CI)	Adjusted OR^a^ (95%CI)
Child born with Hirschsprung’s Disease	13/16.395 (0.08%)	561/1.207.989 (0.05%)	1.71 (0.99–2.96)	1.76 (1.01–3.07)
	SSRI prescriptions redeemed one time only	No prescriptions for SSRIs	Crude OR (95%CI)	Adjusted OR^a^ (95%CI)
Child born with Hirschsprung’s Disease	6/9.663 (0.06%)	561/1.207.989 (0.05%)	1.34 (0.60–3.00)	1.38 (0.61–3.09)
	SSRI prescriptions redeemed a minimum of two times	No prescriptions for SSRIs	Crude OR (95%CI)	Adjusted OR^a^ (95%CI)
Child born with Hirschsprung’s Disease	7/6.732 (0.10%)	561/1.207.989 (0.05%)	2.24 (1.06–4.72)	2.32 (1.10–4.90)

^a^Adjusted for maternal age (≤19, 20–24, 25–29, 30–34, ≥35), maternal smoking (yes/no), sex, of child (male/female), parity (1/<1), calendar year of birth (1996–2001, 2002–2007, 2008–2011, 2012–2016)

## Discussion

The results of our study demonstrated a significantly increased risk of HD after maternal exposure to SSRI. The analyses indicated that only one redeemed prescription for SSRI was associated to a 1.3 fold increased risk of HD, and a minimum of two redeemed prescriptions was associated to a 2.3 increased risk, indicating a dose-response relationship between SSRI and development of HD.

A review of the literature did not yield any previous studies that demonstrated an association between maternal use of SSRIs and HD. The majority of population-based studies examined different groups of congenital malformations [4, 6–10] and none have had specific focus on the association between HD and SSRIs. The aetiology of various congenital malformations differ, and the clustering of gastrointestinal malformations in one group for analyses may render previous studies unable to show a specific association between SSRIs and HD. HD is a rare disease (1:5000 live births) and most of the previous studies may lack the statistical power to find significant associations between maternal use of SSRI and specific malformations [4, 6, 8, 13, 14].

The strengths of the present study were that it was based on nationwide registers and included information on all children in Denmark during the study period. We had no loss to follow up which prevents selection bias. The information on maternal drug exposure was based on prescriptions and not on patient recall therefore eliminating recall bias. Information on the outcome (HD) was collected independently of the exposure status and thereby preventing information bias. In Denmark, HD is treated in one of two national centres and internal review of the HD diagnosis from one of these centres (Odense University Hospital), verified that all HD diagnoses from the NPR was given on the basis of histopathological examination of rectal biopsies or surgical specimens (unpublished data). The validity of the diagnosis of HD is therefore considered high. In our study we had access to a complete nationwide prescription database, ensuring that all mothers could be classified according to the possible prescriptions for SSRIs during the pre-conception period and during early pregnancy. The data in the prescription database are of high quality as previously documented [30]. Furthermore it is strength that we, in sub-analyses, were able to eliminate a possible confounding impact of other maternal underlying diseases that at least in the theory could be associated to congenital malformations. In sub-analyses, we thus excluded women using medications for epilepsy, diabetes, and those who used antipsychotics, anxiolytics and tricyclics antidepressants; and we found that our results were robust as the results from the sub-analyses were similar to our main analyses. With regard to possible confounders, we were able to take into consideration several possible confounders, but the impact of these was limited as the crude and adjusted risk estimates were similar. Overall, the external validity of our study is considered good and applicable to other populations.

Possible limitations of this study were lack of information on drug compliance. We used prescription data as a proxy for drug intake. Previous studies in pregnant women indicated a high rate of compliance in the use of antidiabetic, antibiotic and thyroid medication [31–33]. Patient compliance of antidepressant treatment in Denmark has previously been estimated to 80% [34] and similar findings were found in a Swedish study based on patient interviews (83%) [35]. Any potential misclassification of exposure due to patient non-compliance would, however, tend to underestimate our risk estimates. A high degree of non-compliance could therefore mask a true association. In addition, we did not have enough data to look into the impact of specific types of SSRI. The few number of children born with HD among exposed women did not allow us to give results according to specific types of SSRIs. In a cohort study like this, it may be difficult to isolate the effects of drug treatment, and in case of a statistical significant association (as in our study), it is necessary to consider whether the result is influenced by residual or unmeasured confounding. We had no opportunity to adjust our analyses for severity of underlying depression, and it is unknown whether maternal depression may predispose to HD. An association between major congenital malformation and depression has not been documented [36–38] and HD-associated genes do not match genes associated with depression [39, 40]. Development of the enteric nervous system is dependent on epigenetic stability [41] and maternal depression is associated with epigenetic changes within the DNA of the offspring, including a gene encoding a trans membrane serotonin transporter that regulates the intrasynaptic reuptake of serotonin. This would lead to less intrasynaptic 5-HT, the exact opposite effect of an SSRI, which increases intrasynaptic 5-HT [42]. Other studies did not find epigenetic changes related to maternal depression [43].

HD phenotypes can arise from different genotypes [44] and the genes associated with the most common type of HD (short-segmented HD) are likely to have possible environmental interactions [39, 45]. While discussing confounding by indication in relation to depression and HD it is therefore important to note that depression during pregnancy may be associated with poor nutrition, obesity, smoking, alcohol, and drug abuse [37, 38]. These risk factors can influence the development of the enteric nervous system (causing HD, but the relation between environmental risk factors and HD is not well documented [21, 46]. Maternal smoking has been linked to an increase in gastrointestinal malformations [47], but no association between smoking and HD has been found [48]. One study has reported that vitamin A deficiency (as a proxy for poor nutrition) can impair development of the enteric nervous system and cause a HD-like condition in mice [49]. This is interesting since folic acid supplement compliance during pregnancy is poor especially in young, smoking women with low levels of education [50]. No information on nutritional habits including supplement of vitamins were included in the present study. Maternal obesity may increase the risk of HD [48], but it was not possible to include this information in this study.

## Conclusions

Our data suggest that use of SSRIs in the period of 30 days before conception to end of the first trimester is associated to an increased risk of HD. We cannot rule out that our results might be influenced by unknown confounders, and we do not know whether the increased risk of HD might be associated to specific types of SSRIs. Our results need to be confirmed in other settings, and in the future we need data on a possible impact of specific types of SSRIs. Treatment of depression during pregnancy always has to be weighed against the risks posed by untreated maternal depression during pregnancy, but restrain should be shown in fertile women, who do not use effective contraceptive measures or are planning to become pregnant.

## Additional file

Additional file 1: Table S1. Characteristics of the exposed and unexposed cohorts. All women with prescriptions of antiepileptic, antidiabetic, antipsychotics, anxiolytic and tricyclics antidepressants are excluded from the population. Total number of children = 1,224,384. (DOCX 12 kb)
