# Supplementary material for: Maternal use of selective serotonin reuptake inhibitors during pregnancy is associated with Hirschsprung’s disease in newborns – a nationwide cohort study
Source: Orphanet J Rare Dis. 2017 Jun 20;12:116. doi: 10.1186/s13023-017-0667-4 (PMC5477755; doi:10.1186/s13023-017-0667-4)
Supplement: Additional file 1: Table S1. — Characteristics of the exposed and unexposed cohorts. All women with prescriptions of antiepileptic, antidiabetic, antipsychotics, anxiolytic and tricyclics antidepressants are excluded from the population. Total number of children = 1,224,384. (DOCX 12 kb) [file 13023_2017_667_MOESM1_ESM.docx]

| **Supplementary table:** Characteristics of the exposed and unexposed cohorts. All women with prescriptions of antiepileptic , antidiabetic , antipsychotics , anxiolytic and tricyclics antidepressants are excluded from the population. Total number of children = 1,224,384 | | | | |
| --- | --- | --- | --- | --- |
|  | SSRI prescriptions redeemed a minimum of one time (n=19,807) | SSRI prescriptions redeemed one time (n=9,663) | SSRI prescriptions redeemed a minimum of two times (n=6,732) | No prescriptions for SSRIs  (n=1,207,989) |
| **Maternal age** |  | | | |
| ≤19 | 255 (1.6%) | 155 (1.6%) | 100 (1.5%) | 17,738 (1.5%) |
| 20-24 | 2,183 (13.3%) | 1,294 (13.4%) | 889 (13.2%) | 142,135 (11.8%) |
| 25-29 | 4,798 (29.3%) | 2,762 (28.6%) | 2,036 (30.2%) | 406,438 (33.6%) |
| 30-34 | 5,591 (34.1%) | 3,255 (33.7%) | 2,336 (34.7%) | 427,736 (35.4%) |
| ≥35 | 3,568 (21.8%) | 2,197 (22.7%) | 1,371 (20.4%) | 213,942 (17.7%) |
| **Maternal smoking status** |  | | | |
| No | 11,479 (70.0%) | 6,871 (71.1%) | 4,608 (68.4%) | 926,628 (76.7%) |
| Yes | 4,328 (26.4%) | 2,465 (25.5%) | 1,863 (27.7%) | 180,736 (15.0%) |
| Missing | 588 (3.6%) | 327 (3.4%) | 261 (3.9%) | 100,625 (8.3%) |
| **Sex of child** |  | | | |
| Male | 8,469 (51.7%) | 5,001 (51.8%) | 3,468 (51.5%) | 619,839 (51.3%) |
| Female | 7,926 (48.3%) | 4,662 (48,2%) | 3,264 (48.5%) | 588,150 (48.7%) |
| **Parity** |  |  |  |  |
| 1 | 7,270 (44.3%) | 4,171 (43.2%) | 3,099 (46.0%) | 532,020 (44.0%) |
| >1 | 9,125 (55.7%) | 5,492 (56.8%) | 4,576 (54,1%) | 675,969 (56.0%) |
| **Birth year** |  | | | |
| 1996-2001 | 1,503 (9.2%) | 815 (8.4%) | 688 (10.2%) | 380,913 (31.5%) |
| 2002-2007 | 4,950 (30.2%) | 2,485 (25.7%) | 2,465 (36.6%) | 369,307 (30.6%) |
| 2008-2011 | 5,773 (35.2%) | 3,442 (35.6%) | 2,331 (34.6%) | 233,977 (19.4%) |
| 2012-2016 | 4,169 (24.4%) | 2,921 (30.2%) | 1,248 (18,5%) | 223,792 (18.5%) |
